# Large Scale Proteomic Data and Network-Based Systems Biology Approaches to Explore the Plant World

**DOI:** 10.3390/proteomes6020027

**Published:** 2018-06-03

**Authors:** Dario Di Silvestre, Andrea Bergamaschi, Edoardo Bellini, PierLuigi Mauri

**Affiliations:** Institute for Biomedical Technologies—National Research Council; F.lli Cervi 93, 20090 Segrate, Milan, Italy; andrea.bergamaschi2@studenti.unipr.it (A.B.); edobellini92@gmail.com (E.B.); pierluigi.mauri@itb.cnr.it (P.M.)

**Keywords:** proteomics, systems biology, plants, PPI, network, topology

## Abstract

The investigation of plant organisms by means of data-derived systems biology approaches based on network modeling is mainly characterized by genomic data, while the potential of proteomics is largely unexplored. This delay is mainly caused by the paucity of plant genomic/proteomic sequences and annotations which are fundamental to perform mass-spectrometry (MS) data interpretation. However, Next Generation Sequencing (NGS) techniques are contributing to filling this gap and an increasing number of studies are focusing on plant proteome profiling and protein-protein interactions (PPIs) identification. Interesting results were obtained by evaluating the topology of PPI networks in the context of organ-associated biological processes as well as plant-pathogen relationships. These examples foreshadow well the benefits that these approaches may provide to plant research. Thus, in addition to providing an overview of the main-omic technologies recently used on plant organisms, we will focus on studies that rely on concepts of module, hub and shortest path, and how they can contribute to the plant discovery processes. In this scenario, we will also consider gene co-expression networks, and some examples of integration with metabolomic data and genome-wide association studies (GWAS) to select candidate genes will be mentioned.

## 1. Introduction

During the 20th century, biological research was characterized by Reductionism [1]. By assuming that “the whole is no more than the sum of its parts”, Reductionism states that every biological theory can be deduced by studying the simplest components of biological systems. This dissection has allowed for a listing of the molecules most present in a cell and reveal the complexity of biological systems as well as the limitations of Reductionism itself. In fact, the mathematician and theoretical physicist Jules Henri Poincaré stated that “science is built up with facts, as a house is with stones. But a collection of facts is no more a science than a heap of stones is a house”. In other words, biological systems have emergent properties that cannot be explained or predicted without taking into consideration the molecular interactions that characterize them [2].

**Figure 1:**
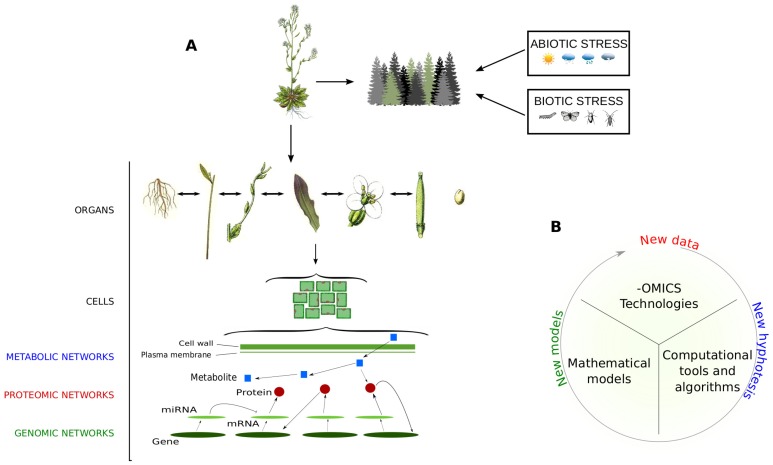
Holistic view of plant organisms. (**A**) Like each living organism, it is assumed that plants are made up of networks integrated and communicating on a multiple scale. They include molecular networks, where genes, transcripts, proteins and metabolites interact to carry out biological functions and processes, and social networks, where plants interact with other organisms/factors and are subjected to abiotic and biotic stresses; (**B**) Steps and main components to perform data-derived systems biology approaches.

Starting from the 21st century, a new concept of investigation called Systems Biology has been adopted to evaluate biological systems from a holistic point of view, by assuming they are made up of molecular networks integrated and communicating on multiple levels [3] (Figure 1). The development of systems biology-oriented approaches is the result of the combination of many scientific disciplines, including biology, mathematics and bioinformatics. In this scenario, important players are the -omic technologies that allow the collection of massive amounts of data faster, efficiently and at reasonable costs [4,5,6]. Mathematical models have been developed to integrate -omic data at a multiscale level [7], while computational tools and algorithms assist biologists in data processing to extract the most relevant information in an objective way [8].

**Figure 2:**
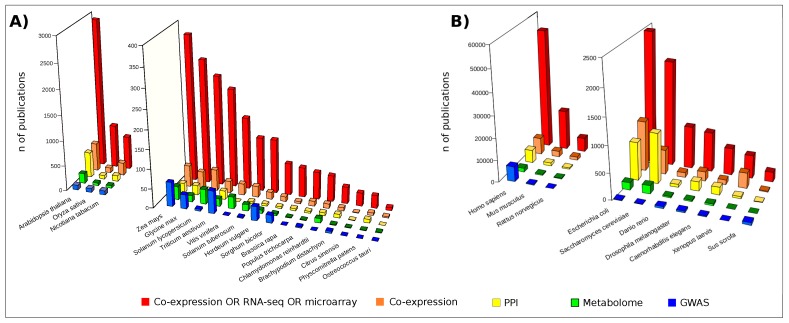
Publications retrieved by interrogating the free search engine PubMED (Data updated in February 2018). Red bars: publications found following the search “Co-expression OR RNA-seq OR microarray [Title/Abstract] AND Organism name”; Orange bars: “Co-expression [Title/Abstract] AND Organism name”; Yellow bars: “Protein-protein interaction [Title/Abstract] AND Organism name”; Green bars: “Metabolome [Title/Abstract] AND Organism name”; Blue bars: “Genome-wide association study [Title/Abstract] AND Organism name”; (**A**) Publications concerning a set of plant organisms; (**B**) Publications concerning a set of model organisms.

The most popular approaches to investigate -omic data at the system level are based on network modeling [9]. While they are already widespread in biomedical [10] and pharmaceutical research [11], their potential to elucidate plant organisms remains, to date, largely unexplored (Figure 2). However, plant biologists are demonstrating their interest and a growing number of studies are addressing the investigation of plant issues, including biotic and abiotic stress, from molecular to systems biology perspectives [12,13]. This landscape of applications is dominated by the use of transcriptomic data usually visualized and analyzed by means of gene co-expression networks [14]. It is noteworthy that some studies based on integrative strategies combined transcript and metabolic profiles with genome-wide association studies (GWAS) [15,16,17] and quantitative trait loci (QTLs) data [18,19]. On the contrary, fewer studies relied on the combination of high-throughput proteomic data and protein-protein interaction (PPI) network models [20,21,22]. However, in the last few years an increasing number of authors focused their activity on the high-throughput profiling of plant proteomes [23,24,25,26,27,28,29,30] as well as on the experimental [31,32,33] and computational detection of PPIs [34,35,36,37].

Based on these premises, our review aims to explore the state of the art and the perspective of -omic technologies and systems biology-based approaches in contributing to elucidate the biological mechanisms underlying plant traits. Special attention will be given to the potential that may derive from the topological analysis of co-expression and PPI network models [38]. Although to date few studies of this type have been carried out in the context of plant biology, some examples well foreshadow the benefits that these approaches may provide to plant research and they will be mentioned in greater detail.

## 2. Omic Technologies in the Plant World: From Genomics to Metabolomics by Way of Proteomics

**Figure 3:**
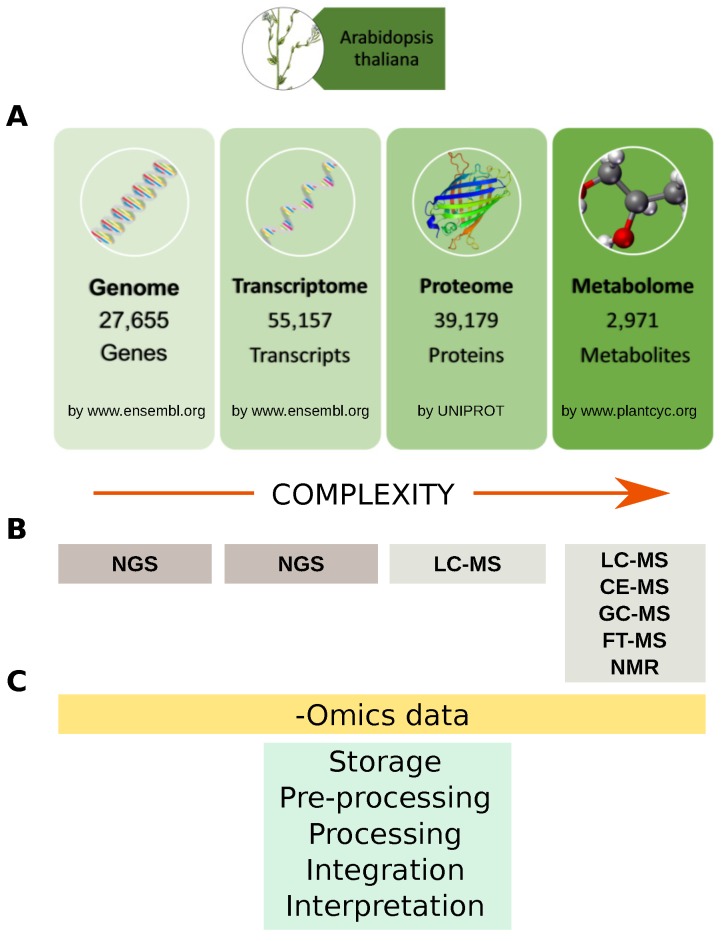
Omics data and analytical technologies. (**A**) Example of *Arabidopsis thaliana* genes, transcripts, proteins and metabolites available in specialized databases; (**B**) Main analytical technologies for profiling genes, transcripts, proteins and metabolites. Data complexity increase from genome toward metabolome. To date about 39,179 (more than 23,000 are unreviewed) *Arabidopsis thaliana* proteins are stored in the UNIPROT database, while it is estimated that plant kingdom metabolome counts more than 200,000 metabolites with a vast range of functional and structural diversity; (**C**) Main steps for handling-omics data from storage to biological interpretation. NGS: Next Generation Sequencing; LC: Liquid Chromatography; CE: Capillary Electrophoresis; GC: Gas Chromatograpgy; FT: Fourier Transform; MS: Mass Spectrometry.

A system-wide understanding of the molecular mechanisms underlying biological phenotypes has been achieved by the increasing surge of -omic data, both new and already available in public repositories. Data-derived systems biology approaches aim to use these data to infer new models or to integrate them into existing ones, with the purpose of formulating new hypotheses to be tested [39]. They may be basically clustered in those that formulate models starting from experimental data, e.g., co-expression network [40], gene regulatory networks [41], protein-DNA network [42], and those that integrate experimental-omic data on existing models, e.g., pathways [43], protein-protein interaction (PPI) network [44]. Ideally, multi-omic approaches have the advantage of revealing different domains of gene function. At the same time, the hypotheses formulated by multiple independent data sources are more likely to be robust and true. Thus, the outcome of data-derived systems biology approaches is closely related to the availability of molecular profiles listing genes, transcripts, proteins and metabolites (Figure 3).

An overview of the main analytical technologies will be provided in the following subsections and special attention will be given to proteome and PPIs profiling. Although this scenario is characterized by bioinformatic procedures whose role is not marginal for the performance of these approaches, a detailed description of tools and algorithms to assist researchers in -omics data processing is not the focus of this review. However, a representative list reporting some tools mentioned in the text is provided (Table S1).

### 2.1. Genomics

**Figure 4:**
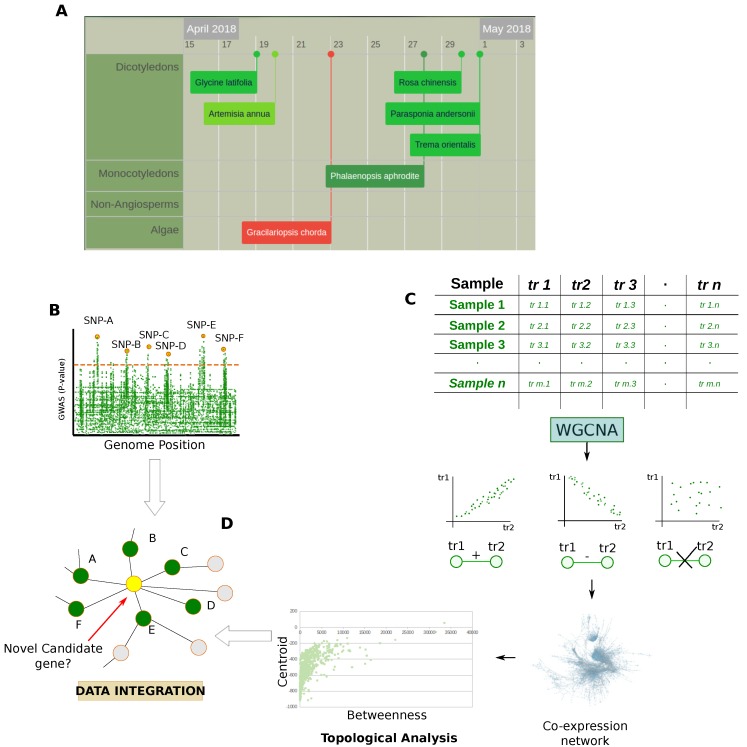
Genomic and transcriptomic data. (**A**) Snapshot from Plabi database (http://www.plabipd.de) showing an interactive display of all plants which have had their genomes sequenced to date. Additional data such as publication data, genome size and a link to the paper is provided for all genomes; (**B**) GWAS are observational studies that correlate a set of specific variants (single-nucleotide polymorphisms, SNPs) in a population with phenotypic traits. In the case of QTL studies, specific genome positions are put in relationship with quantitative traits; (**C**) Matrices of transcriptomic data are processed by statistics that measure the dependence between variables e.g., Pearson’s correlation, Spearman’s correlation, and Mutual information, to model them in the form of co-expression network which is subsequently evaluated at a functional and topological level; (**D**) Integration of results obtained by the topological evaluation of the co-expression networks with the genetic architecture of genes provided by GWAS and QTL studies.

Despite the plant’s genome size and their complex ploidy, Next Generation Sequencing (NGS) techniques are having an impact on plant research and more and more genomes are decoded (Figure 4A). Although microarrays are still widely used, especially in meta-expression analysis studies, these technologies are revolutionizing genomic and transcriptomic studies. In fact, NGS allows the sequencing of a whole genome rapidly and at relatively low cost, also providing measurement of different RNA populations (siRNA, miRNA, mRNA), unknown sequences, alternative splicing and mutations [45]. These improvements are having a strong impact on the analysis of non-model organisms, including plant ones. In fact, in addition to technical issues related to chemical hybridization, e.g., cross-hybridization, non-specific hybridization, and limited detection range of expression, the use of microarrays is limited to organisms with available genome sequences [46].

In addition to positively affecting the feasibility of the high-throughput proteomic analysis for organisms poorly sequenced at the proteomic level, the availability of NGS techniques gives the opportunity to perform genome-wide association (GWAS) [47] and quantitative trait locus (QTL) studies [48] (Figure 4B). These approaches are widely used in plant biology and a particular interest lies in investigating crop species [47]. For example, recent studies characterized genetic networks underlying agronomical traits in soybean [49], while others adopted these approaches to identify functional associations between genes and metabolism in *Arabidopsis thaliana* [15,16,17].

In comparison to proteomic and metabolomic technologies, genomics development remains surely ahead. In fact, thanks to technologies like microarrays, data-derived systems biology was first conceived to evaluate transcriptomic data [9]. A popular approach widely used relies on statistics that measure the dependence between variables [50], modeling the experimental data in the form of co-expression networks that are topologically evaluated (Figure 4C). Of note, some authors combined the topological evaluation of co-expression networks with the genetic architecture of genes and signatures provided by GWAS and QTL studies [19,51,52]. The integrative approaches used in these studies allow a more robust formulation of hypotheses as well as the possibility to improve the discovery of new candidate genes (Figure 4D). At the same time, the availability of multi-omic data allows for the elucidation of different aspects of the gene function.

### 2.2. Proteomics

Plant proteome analysis has been historically performed by two-dimensional gel electrophoresis (2DE) or 2-D Fluorescence Difference Gel Electrophoresis (2D-DIGE) which carry a series of drawbacks, ranging from the laborious protocols to the poor resolution of underrepresented proteins, with extreme pI, MW or highly hydrophobic [53]. Many of these limitations were improved over the years and many laboratories still use these methodologies. However, the evolution of proteomic technologies based on the combination of liquid chromatography (LC) and mass spectrometry (MS) [54] is strongly attracting the attention of plant biologists, and some topics, including tissue profiling [27,55], stages of development [30,56] and proteome modulation between physiological and stress conditions [23,24], have been already addressed.

**Figure 5:**
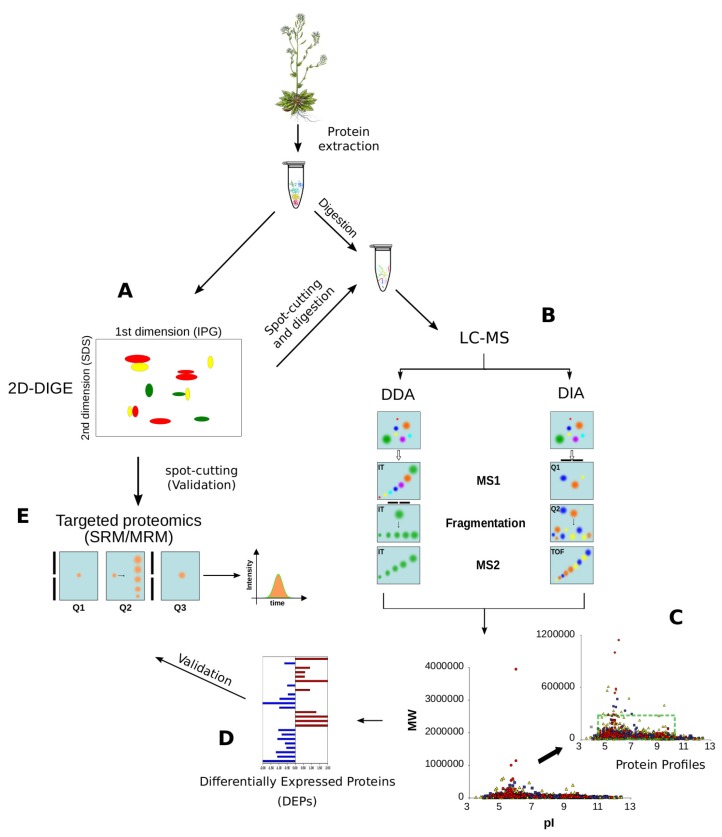
Proteomic methodologies to analyse plant proteomes. (**A**) 2DE (2-Dimensional Electrophoresis) and 2D-DIGE (2 Dimensional Fluorescence Difference Gel Electrophoresis) are the most popular approaches to resolve and quantify plant proteins, respectively; (**B**) After spot-cutting, protein identification is usually performed by LC-MS; (**C**) Data Dependent Acquisition (DDA) and Data Independent Acquisition (DIA) approaches are demonstrating a great capability to identify thousands of proteins per sample, without any limitation in pI, MW or hydrophobicity; the green box shows the limits of 2DE resolution; (**D**) At the same time, the DDA and DIA approaches allow the quantification of hundreds of proteins simultaneously by label- and label-free approaches; (**E**) SRM/MRM is the elective method to validate protein quantitations by MS and it is often applied to confirm the differential expression discovered by 2D-DIGE or shotgun proteomics. Selection of the parent ion occurs in the first mass analyzing quadrupole (Q1), which is set to a narrow mass window according to the masses of the ion(s) of interest. Collision induced disassociation in the second quadrupole (Q2) yields the fragmentation of the parent ion in product ions which are detected in the third quadrupole (Q3) which is set to an appropriate narrow mass window. When Q3 is replaced by Q-Orbitrap we refer to parallel reaction monitoring (PRM).

Current high-throughput proteomic methodologies based on LC-MS can be grouped into shotgun and targeted proteomics, or based on their data acquisition mode e.g., Data Dependent Acquisition (DDA) and the more recent Data Independent Acquisition (DIA ) (Figure 5). The advances in LC separation and MS instrumentation are providing more and more high analytical reproducibility, speed in acquisition, high-resolution and sensitivity up to femtomoles [57,58,59]. The improvement of these aspects, coupled with sample preparation protocols [60] and advanced bioinformatic tools [61], may be translated into higher plant proteome coverage, including post-translational modifications (PTMs) and PPIs [62]; PTMs identification often requires specific enrichment steps, like in the case of phosphorylation or glycosylation, which complexify sample preparation procedure as well as protein quantitation [63]. To reach these purposes, a key factor is the massive application of the high-throughput genomic technologies in order to increase the number of sequenced genomes, including that of the crop species [64]. The availability of genomic sequences is fundamental in the application of high-throughput proteomic technologies for the investigation of non-model plants [65]. In fact, they enable the use of algorithms and software to process and interpret the huge amount of experimental tandem mass spectra (MS/MS) produced per experiment [66]. Of note, spectra interpretation can now be performed by De Novo sequencing algorithms that do not require the availability of reference sequences [67]. They provide the opportunity to improve the proteomic analysis of non-model plant organism [68] and open new perspectives in profiling and validating gene expression at the protein level [69].

#### 2.2.1. Shotgun Proteomics

Shotgun proteomics aim to identify proteins by analyzing a mixture of peptides through a combination of high performance LC and tandem mass spectrometry (MS/MS) [70]. When peptides are separated through on-line two dimensional HPLC, prior to MS/MS analysis, we refer to Multidimensional Protein Identification Technology (MudPIT) [26,71]. Recent studies on plants confirmed that the MudPIT approach is an excellent tool for both qualitative and quantitative proteomic analyses. Thousands of proteins and hundreds of differentially expressed proteins were identified in leaves [23,25], calli [55] and calli’s secretome [72] from rice. Large scale profiling was also performed on the *Arabidopsis thaliana* floral proteome [73]. As for the application of the MudPIT approach to non-model organisms, Patel et al. identified 2358 proteins by analyzing the eukaryotic microalga *Chlamydomonas reinhardtii* in response to a nitrogen source [74], while Islam et al. identified a total of 4178 proteins by investigating the nitrogen remobilization in poplar [27]. In our laboratory experience we adopted the MudPIT approach to examine dynamic changes in the protein composition during salt-stress adaptation in microsomes from *Mesembryanthemum crystallinum* leaves [71], while more recently we investigated the early physiological, ionomic and biochemical changes occurring in *Cucumis sativus* roots and leaves under single or combined Mo and Fe starvation [26]. As for *Mesembryanthemum crystallinum* we contributed in investigating the majority of the subunits of V-ATPase during the salt-induced transition from C3 to crassulacean acid metabolism (CAM). In addition to identify and quantify these membrane proteins, usually difficultly characterized by means of 2DE, we found some glycolytic enzymes, such as enolase and phosphoenolpyruvate carboxylase 1, assuming their membrane association with subunits of the vacuolar H(+)-ATPase V-ATPase as previously suggested [75]. In the case of *Cucumis sativus* the identification of thousands proteins (>1400) and hundreds proteins differentially expressed highlighted the central role of mitochondria in the coordination of Fe and Mo homeostasis and allowed us to propose the first model of the molecular interactions between these elements. Moreover, it represents one of the first studies concerning the large scale protein profiling of mitochondria purified from roots of *Cucumis sativus*, and the simultaneous identification and quantification of hundreds of proteins was a plus over previous similar works conducted with 2DE [76].

All studies mentioned in this section highlight the great capability of the MudPIT approach to provide a good snapshot of the analyzed proteome in terms of proteins identified and differentially expressed. In the case of non-model organisms, these profiles represent an important starting point to verify thousands of predicted proteins as well as their PTMs. Unfortunately, it implies also that the functional evaluation of these huge amounts of data is often limited due to the lack of well-defined annotations, including PPIs. For example, only 124 out of 24,835 *Cucumis sativus* proteins stored in UNIPROT database are at the moment manually annotated by means of a critical review of experimentally proven or computer-predicted data, while this procedure, to date, has been applied to less than 40% of *Arabidopsis thaliana* proteins (Figure 3).

#### 2.2.2. Selected- and Multiple-Reaction Monitoring (SRM/MRM)

Since shotgun proteomics preferably fragment high-abundant precursor ions, dynamic range and under-sampling issues represent their main limitations. In fact, although these phenomena could be attenuated by performing biological and technical replicate analyses, they introduce a certain element of randomness into peptide detection, impacting both protein identification and quantification [77]. A more accurate protein quantitation is assured by targeted proteomic approaches, e.g., selected- or multiple-reaction monitoring (SRM/MRM) mass spectrometry (MS) [78]. A triple quadrupole mass spectrometer is usually used to monitor, fast, quantitatively and cheaply, a set of predefined peptides in a complex mixture resulting from the enzymatic digestion of a protein sample. The need to know a priori the so-called “transition” to specifically monitor those peptides, and thus the related parent proteins, represents a limitation of these approaches and may explain their poor use to quantify plant proteins. Nevertheless, due to the increasing application of shotgun proteomic approaches we expect that an increasing number of MS spectra concerning plant proteins will be stored in specialized databases to be computationally processed for assisting in the design of best transitions [79]. Differently, a variant of this approach, called parallel reaction monitoring (PRM), is based on Q-Orbitrap as the representative quadrupole-high resolution mass spectrum platform [80]. It is most suitable for quantification of multiple proteins with an attomole-level detection as well as in assay development for absolute quantification. In fact, by means of PRM the linear range increase to 5–6 orders of magnitude and the mass accuracy can reach to ppm level. In addition, since a full MS/MS spectra acquisition is performed, the selection of the ion pair and the optimization of the fragmentation energy are not necessary [81]. However, although the MS/MS spectra from PRM can be searched directly with traditional database search engines, a spectral library with high quality reference MS/MS spectra is essential for targeting and quantifying peptides, thus for the success of PRM [82].

Recent applications of SRM/MRM to quantify plant proteins concern the proteome variation in strawberry fruit at different ripening stages [83] and in tomato during the formation of tomato fruit cuticles [84], while it was used to monitor phosphorylation targets in relation to symbiotic signaling in *Medicago truncatula* [85]. Some authors adopted SRM/MRM to quantify food allergens and contaminants highlighting the effectiveness of this approach in this field of application [86,87]. On the other hand, the limited number of transitions that may be followed per experiment make SRM/MRM a method to validate candidate markers rather than to identify differentially expressed proteins at a large scale level (Figure 5). As a consequence, its use in plant biology may be more relevant in providing an alternative to validate protein quantitation avoiding costs due to antibody development [78], an aspect particularly helpful for non-model plant species that most suffer from the lack of specific immunoreagents.

#### 2.2.3. DIA/SWATH

Sequential window acquisition of all theoretical fragment ion spectra (SWATH) is a new approach, based on DIA, introduced to extend the proteome coverage of shotgun proteomics, based on DDA, and the degree of multiplexing achieved by SRM/MRM. It relies on isolation and fragmentation of all the precursor ions within a defined “mass window”, called SWATH. Thus, it should allow the monitoring of all peptides present in a complex biological sample improving the proteome coverage and its quantitation [88].

Similarly to SRM/MRM, a library containing experimental acquired spectra is necessary for the bioinformatic analysis of DIA/SWATH data [82]. Since most tools and databases yielded are limited to the most popular model organisms, e.g., human, yeast and mouse, it represents a drawback in investigating plant proteomes by DIA/SWATH. To overcome this limitation, Fan et al. used a web-application called MRMaid that exploits millions of identified peptide spectra held in PRIDE database [79]. By means of MRMaid, the authors designed optimal transitions for 25 *Arabidopsis thaliana* proteins, and 23 of them were correctly quantified and validated. A spectral library of more than 5000 proteins was experimentally built for *Solanum lycopersicum* and optimized to be best suited in processing of DIA/SWATH data acquired on TripleTOF instruments [89]. A prior generation of a spectral library based on DDA data was also performed by Zhu et al. to investigate inferior and superior rice spikelets during grain filling [90] and to understand the mechanisms underlying the molecular response of *Arabidopsis thaliana* to lead pollution [29]. In *Arabidopsis thaliana* they identified and quantified the expression of 1719 proteins in water- and Pb-treated plants, and 231 proteins showed significant abundance changes upon Pb exposure. This study is one of the first examples of the application of DIA/SWATH analysis in *Arabidopsis thaliana*. The potential for this approach to investigate plant organisms has yet to be fully exploited. Moreover, the great fitness in identifying and quantifying thousands of proteins per experiment makes DIA/SWATH data ideal to be used in strategies of investigation based on data-derived systems biology. Thus, in the next few years, we expect a growing number of plant proteomic studies based on this approach.

#### 2.2.4. MS-Based Quantification Strategies

It is well established that biological functions are rarely attributed to individual molecules, thus the characterization of single biomarkers could lead to incomplete representation of real models. To quantify specific proteins, antibody-based methods, such as enzyme-linked immunosorbent assay (ELISA) and western blotting, have been used for decades. Unless arrays are adopted [91], their fitness to quantify proteins at a large scale level is poor. Conversely, MS-based quantification allows the identification of hundreds of proteins differentially expressed (DEPs) by both label [92] and label-free [93] approaches. Thus making MS proteomic data a good source for data-derived systems biology to investigate plants in a holistic way.

##### Label-Free Quantitation

**Figure 6:**
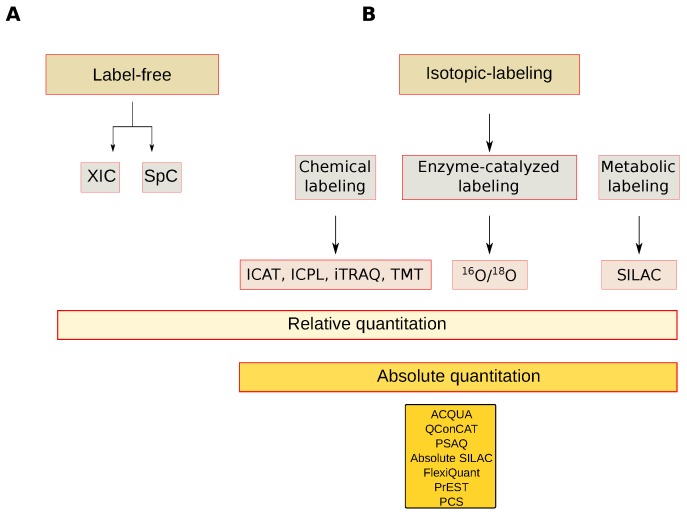
Mass spectrometry-based quantitative approaches. (**A**) Label-free approaches; (**B**) Isotopic labeling approaches. XIC: Extracted-ion chromatogram, SpC: Spectral count, ICAT: Isotope-coded affinity tag, ICP: Isotope-Coded Protein Label, iTRAQ: Isobaric tag for relative and absolute quantitation, TMT: Tandem Mass Tags, ^16^O/^18^O: Oxygen isotope ratio, SILAC: Stable isotope labeling by/with amino acids in cell culture, AQUA: Absolute quantification, QConCAT: Quantification concatamer, PSAQ: Protein Standard Absolute Quantification, Flexiquant: Full-length expressed protein quantification standard, PrEST: Protein Epitope Signature Tags, PCS: Peptide-Concatenated Standards.

The most common MS-based quantification strategies are classified in label-free and stable isotope-labeling, and the latter may be further grouped into relative and absolute protein quantification approaches [94] (Figure 6). Label-free quantitation is a simple and low-cost alternative to relatively quantify proteins at a large scale level. Quantitation is done by comparing the chromatographic peak area of extracted ions (XICs) or spectral count (SpC) values. Chromatographic peak area evaluation requires high analytical reproducibility to correctly align the precursor ions and avoid quantitation errors which are more frequent when MS signals to be compared derive from different experiments or are from a different instrumental set up. However, dedicated bioinformatic algorithms have been developed to minimize these experimental variations with the purpose of improving data reproducibility [95].

In comparison to chromatographic peak integration, SpC based quantitation is most popular because it uses a simpler procedure and it can be accommodated to any type of organism and most workflows [96]. In fact, since it is based on the empirical observation that more peptides correspond to more protein, the relative protein quantitation is simply obtained by counting and comparing the total number of MS/MS spectra associated with all peptides detected for the corresponding parent protein. The outcome derived from this procedure may be affected by the MS analytical reproducibility especially when dealing with shotgun proteomics, due to the undersampling resulting from the stochastic nature of the DDA method. Thus, prior to comparing SpC values, adjustment by data normalization strategies may be necessary [97].

To infer expression changes by means of SpC comparison, several methods have been implemented in recent decades and the most popular include the statistical G-test [98], the normalized spectral abundance factor (NSAF) [99] and the protein abundance index (emPAI) [100]. Their effectiveness in discovering reliable differentially expressed proteins was also confirmed in the context of plant proteomics [26,71,101,102,103,104,105]. The robustness of the results shown in these studies was assured by replicate analyses and by a careful planning of the samples analyzed in order to reduce systematic and non-systematic instrumental variations. These needs are minimized when labeling techniques are used and samples to be compared are pooled limiting technical variability.

##### Isotope-Labeling Quantitation

The most popular labeling approaches to quantify proteins include isotope-coded affinity tag (ICAT), used by different authors to characterize S-nitrosylated proteins in *Arabidopsis thaliana* [106,107]. Although the ICAT method reduces sample complexity at the peptide level, sample preparation makes it complex and demanding. In addition, only cysteine-containing peptides are analyzed, impairing the quantification of those that do not contain this aminoacid. Many more studies to quantify plant proteins have adopted isobaric tags for relative and absolute quantitation (iTRAQ) [108,109,110] and tandem mass tags (TMT) [111]. In comparison to ICAT, or ICPL [112], TMT and iTRAQ labeling takes place at the peptide level by means of amine specific reagents which assure a wider peptide labeling without loss of PTM information. The power of iTRAQ was recently described by Vélez-Bermúdez et al. who systematically quantified more than 12,000 *Arabidopsis thaliana* proteins by adapting an experimental protocol for plants [113]. On the other hand, a drawback of these approaches is related to the co-elution of peptides that, isolated within the same precursor ion window, may cause systematic read-out errors. Therefore, TMT and iTRAQ quantitation benefit from high-resolution precursor ion selection and extended protein and peptide fractionations.

In addition to chemical labeling, peptides may be tagged enzymatically, e.g., ^16^O/^18^O [114], and metabolically, e.g., SILAC [115]. Although simple modifications in SILAC protocol enable in plants similar quantitation accuracy, precision and reproducibility as in animal cells, the application of SILAC to quantify plant proteins remain challenging. In fact, under their natural growth conditions, plants could not be fully labeled with stable isotope-coded amino acids. An alternative metabolic labeling strategy based on inorganic nitrogen isotopes was developed [116]. Although in terms of sample preparation and costs this procedure remains demanding, it provides reproducible and accurate results. Of note, this new protocol gives the possibility to grow plants in hydroponic solutions, which may allow a tight control over nutrient uptake.

##### Absolute Quantitation

Procedures and strategies so far discussed, both label or label-free, usually refer to the relative quantitation of proteins from different tissues, compartments or different functional cellular states. In addition to proteins that change their expression between different conditions, it is often useful to estimate their absolute quantification [117]. In recent years, several methods for absolute quantitfication have been proposed. From our knowledge, only absolute quantification (AQUA) [118] and quantification concatemer (QConCAT) have been used to absolutely quantify plant proteins. However, other methods include absolute SILAC [119], FlexiQuant [120], protein epitope signature tag (PrEST) [121] and peptide-concatenated standards (PCS) [122] (Figure 6).

All absolute quantification methods are a technical challenge, since they rely on the use of a spiked-in reference standard and can only be performed on a small number of proteins per experiment. Nevertheless, they provide relevant information for biomedical applications e.g., biomarkers in body fluid, and to estimate the cellular protein copy number. For this purpose, knowledge of the number of cells used for the analysis, spike-in reference and proteomic analysis are required [123]; alternatively, some authors proposed that MS-signal of histones can be used as an internal standard since it is proportional to DNA, thus avoiding the need to use spike-in standards [124].

In addition to being used for simulation processes in systems biology studies, absolute quantification has been associated with network topology [125]. Heo et al. found that interaction between two proteins depends not only on their binding affinity but also on their concentrations. It is worth noting that the authors postulated that intracellular abundances of proteins evolve to anticorrelate with node degrees in the network, thus suggesting that the control of protein abundances may represent an important factor in the design and evolution of natural PPIs.

### 2.3. Metabolomics

As for plant research, genomic, transcriptomic and proteomic approaches may be considered quite new, while those used to identify and quantify metabolites have a longer history. In fact, despite the fact that plant metabolome remains largely to be explored due to its complexity, metabolomic studies have contributed in understanding plant biology from the view of metabolites that, along with proteins, reflect the endpoint of most biological activities and provide a functional screen of the cell physiology [126].

To date, it is estimated that plant kingdom metabolome counts more than 200,000 metabolites with a vast range of functional and structural diversity [127]. A major database containing plant metabolites is the Plant Metabolomic Network Database (PMN) [128]; it collects data from 76 different species showing pathways, enzymes and reactions. Organism-related projects have been developed for tomato [129], soybean [130] and *Arabidopsis thaliana* mutants [131], while databases containing plant metabolomic profiles obtained by specific analytical technologies have been built for NMR data, e.g., MeRy-B [132]. Due to the plant metabolome complexity, its systematic analysis should be addressed by means of the use of complementary methodologies of extraction, identification and quantification [133,134]. However, metabolomic platforms offer coverage of just 10% of the small molecule complement of the cell, thus further efforts will be necessary to obtain more comprehensive metabolomic profiles [135]. In fact, in combination with genomic and proteomic data, a global view of primary and secondary metabolites may help plant biologists to move toward a systems-level understanding of plant physiology [136], impacting a broad range of topics including plant growth, stress responses and crop quality improvement [137].

The field of plant metabolomic analysis was pioneered by Magnetic Resonance Nuclear (NMR). It has greatly contributed to investigating primary and secondary metabolites in the context of topics ranging from food traceability [138] to plant response to abiotic [139] and biotic stresses [140]. To date other technologies e.g., matrix-assisted laser desorption/ionization (MALDI)–MS for metabolite imaging [141], Fourier transform ion cyclotron resonance mass spectrometry (FT-ICR-MS) [142] and MS coupled with different systems of separation (LC, gas-chromatography (GC) and capillary electrophoresis (CE )) [143], are available to perform plant metabolomic analysis at an advanced level. The major advantage of MS is its high sensitivity. LC-MS was used in profiling both primary and secondary metabolites [144,145]; hydrophilic interaction liquid chromatography (HILIC) coupled to MS represents an attractive complementary tool to analyze highly polar/ionic plant metabolites [146]. The use of GC-MS is instead limited to thermally stable volatile compounds, making the analysis of high molecular weight compounds difficult [147]. Nevertheless, it is a reproducible and sensitive approach widespread in the context of plant metabolism [148,149]. High-resolution and high mass accuracy is assured also by FT-MS, which facilitates structural characterization [150], and by CE-MS whose major advantage is the possibility of analyzing almost any charged species by both cationic and anionic methods [151]. Finally, more recently, targeted metabolomic approaches have begun to gain ground pointing to the analysis of a pre-defined set of metabolites and their absolute quantification [152].

## 3. Co-Expression and PPI Networks as Models to Investigate Plant Organisms

To objectively extract meaningful information from the huge amount of -omic data provided by the advances in genomic, proteomic and metabolomic technologies, strategies based on graph theory are demonstrating to be helpful and their use is rapidly increasing. The main adopted procedures are basically divided into two groups: those that formulate models starting from experimental data, e.g., co-expression network [14], gene regulation networks [153], protein-DNA network [154], and those that integrate experimental -omic data on existing models, e.g., pathways [43], protein-protein interaction (PPI) networks [20]. In this scenario, the most popular approaches to globally investigating transcript and protein profiles rely on PPI and co-expression network models. These molecular profiles are usually processed at a functional level through the gene ontology term enrichment, and several tools have been developed for proteins, e.g PSEA [155], and genes e.g., GSEA [156], respectively. At the same time, the topology of co-expression and PPI network models is analyzed by means of specific algorithms to identify the most relevant topological molecules [38]. In fact, it is demonstrated that network structure is informative and its evaluation may help biologists in understanding the biological issues addressed [9].

### 3.1. Gene Co-Expression Networks

The strategies to evaluate biological systems in a holistic way ideally aim to understand their emergent properties by integrating different kinds of -omic data and by taking into consideration their functional and molecular relationships. A representative example taking into account all of these aspects concerns the reverse engineering of gene regulatory networks (GRN), where pairs of genes are considered in a systemic perspective of cooperation, including co-regulation, activation/suppression, and indirect control through the action of siRNA, miRNA, proteins, metabolites or epigenetic mechanisms [153]. The level of inference required by these approaches makes necessary the availability of a wide range of information, as well as modeling techniques e.g., Boolean networks, Bayesian networks or differential equations (ODEs), that increase the complexity of these methods and restrict their application to sparsely connected networks of small sizes [50].

Co-expression networks are defined as undirected graphs where nodes correspond to transcripts and edges indicate their meaningful dependence (Figure 4). They are part of the reverse engineering approach, but unlike GRN the direction and type of co-expression relationships are not determined [157]. Co-expression became popular to evaluate the surge of data provided by microarray and RNA-Seq technologies. The idea behind this processing is based on the assumption that co-expressed genes are controlled by the same transcriptional regulatory program, functionally related, or members of the same pathway or protein complex. Thus, transcript profile of time series, or following specific perturbations, may be indicative of dynamics and differences between transcripts, implying their regulation [158].

Co-expression networks were recently used in plants to investigate the response to abiotic stresses [159], tomato fruit ripening [160] and the response to concentrations of nitrogen in maize [161]. To process trancriptomic data and reconstruct the co-expression network, the most used statistics include Pearson’s correlation (PC), Spearman’s correlation, Kendall’s correlation and mutual information. For major details about tools and strategies applied to perform such analyses in plants, we refer to recent reviews [162]. To build and analyse gene co-expression networks, weighted correlation network analysis (WGCNA) is among the most popular tools and it has also been recently adopted to process plant transcript level [163,164]. Specifically, Qiao et al. re-analyzed sweet orange fruit transcriptome data leading to the identification of 72 genes highly correlated with the fruit sugar/acid ratio, while Tan et al. adopted an integrative approach to identify DEGs and network modules in response to various cadmium stresses in rice root. The topological network analysis also exploited by Zhaoming et al. to investigate soybean seed development [165]. By means of WGCNA, they uncovered 46 different modules of gene expression patterns and seven hub genes were identified as being involved in soybean oil and seed storage protein accumulation processes. Similarly, a tool called SWIM [166] was recently developed and applied to investigate the transcriptional changes underlying berry formation and ripening in 10 varieties of grapevine [167]. Massonnet at al. identified the core transcriptome of berry development, the transcriptional differences between red and white berry as well as of common transcriptomic traits. In addition to highlighting the propensity of co-expression network based approaches in visualizing and analysing the large amount of data produced by microarray and RNA-seq experiments, these studies confirmed the effectiveness of network topological analysis in extracting relevant molecules and hub genes representing a foundation for further research.

#### Gene Co-Expression Network Combined with GWAS and QTL Data

Most studies based on transcriptomic data and gene co-expression share the topological analysis of the reconstructed networks to identify modules and hubs related to the investigated biological issues/phenotypes [163,164,167]. The selection of candidate genes or the identification of phenotype-associated subnetworks and pathways is also driven by GWAS and QTL data that are imposed as a starting point in network analysis (Figure 4). In addition to evaluating how information is propagated through a network of interacting molecules, these strategies aim to integrate information from population-based and molecular profiling studies to support the selection of true phenotype-associated genes. In fact, genetic variants are often distributed across several genes and this genetic hetereogeneity restricts the phenotype-associated variants to subpopulations reducing the statistical power of association [168]. To overcome this limitation and to reduce the loss of true phenotype-associated genes, a web application called araGWAB integrates GWAS data and co-functional network information [52]. Similarly, Kobayashi et al. reconstructed a co-expression network to independently verify if the genes selected by GWAS were correlated in contributing to salt tolerance in *Arabidopsis thaliana* [51]. In this context of association between population-based and molecular profiling data it is interesting to note the topological evaluation of the co-expression network and its relationship with the genetic architecture of genes and signatures provided by GWAS and QTL studies. For instance, Mahler et al. found that genes associated with QTLs were underrepresented in the network module core, while there was a higher representation in the periphery of the co-expression network [19]. In addition, high-connected genes (hubs) showed a lower level of polimorphism suggesting they are buffered against a large expression modulation and that network topology may influence gene expression and sequence evolution.

The integration of complementary approaches and data is a strategy that improves a reliable identification of genes associated with a given phenotype. However, the quality of reconstructed co-expression networks and the availability of complete annotations may strongly influence this selection. It also concerns *Arabidopsis thaliana* which represents the ideal organism for GWAS studies due to its inbreeding nature [169]. In fact, although ARANET is one of the most comprehensive network databases of Arabidopsis thaliana, it still falls short in the complete reconstruction of biological processes. Moreover, only about 40% of Arabidopsis thaliana protein-coding genes stored in UNIPROT have manually-annotated records with information extracted from literature and curator-evaluated computational analysis, while this percentage collapses for most non-model organisms and crop plants, including *Oryza sativa* (8%), *Zea mays* (<1%) and *Vitis vinifera* (<1%), whose annotations are mainly predicted or unknown [162].

### 3.2. Protein Co-Expression Networks

Measures of dependence between variables have recently been used to abstract high-dimensional proteomic data in the form of protein co-expression networks [170]. Similarly to microarrays and RNA-seq, MudPIT or DIA/SWATH data are multi-dimensional and may be formatted in a m × n matrix with the result that they are processed by means of algorithms and strategies typically used for analyzing genomic data [9] (Figure 4). When these strategies are applied to proteomic data they need to be properly modified because proteomic datasets are often incomplete and a major issue concerns the high rate of missing values that introduce loss of information and significant bias [171]. In addition, appropriate data preprocessing or proper thresholds of filtering are prerequisites to capture true correlations [172].

The use of co-expression-based approaches to process high-dimensional proteomic data is yet largely unrealized and to date few studies are published [9]. To our knowledge no studies of this type have been conducted on plant organisms. However, protein co-expresson could allow the identification of relevant molecules in a complementary way with respect to the most popular quantitative analysis or network approaches based on the integration of experimental data and the PPI network model. Of note, the opportunity to abstract and analyse at the network level non-model organisms should not be underestimated, including plants, whose interactome is incomplete or more often not available.

### 3.3. PPIs Identification

**Figure 7:**
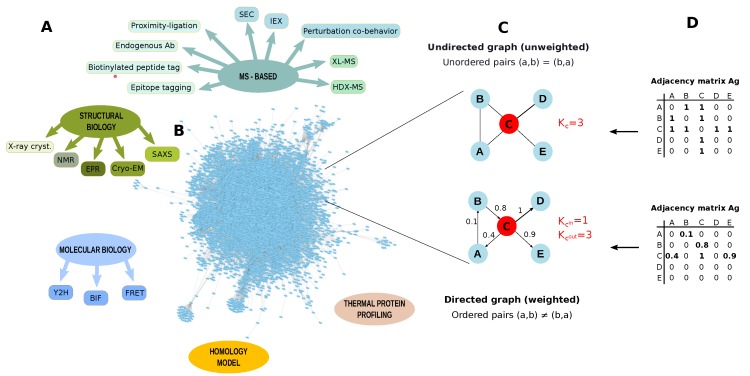
Protein-protein interactions (PPI) identification. (**A**) Methodologies to identify protein-protein interactions (PPI); (**B**) Arabidopsis thaliana PPI network retrieved from STRING database by applying a threshold of 0.7. The network contains 8857 nodes and 180,178 edges (Experimentally validated/Database); (**C**) Example of graph undirected and unweighted and directed and weighted; (**D**) Adjacency matrices describing a graph undirected and a graph unweighted and directed and weighted. PPI networks are usually unweighted and undirected. K = node degree, K^in^ = node in-degree, K^out^ = node out-degree, SEC: Size-exclusion chromatography, IEX: ion exchange chromatography, XL-MS: cross-linking mass spectrometry, HDX-MS: Hydrogen Deuterium Exchange mass spectrometry, X-ray cryst.: X-ray crystallography, NMR: Nuclear magnetic resonance, EPR: Electron paramagnetic resonance, Cryo-EM: Cryo-electron microscopy, SAXS: Small-angle X-ray scattering,Y2H: yeast two-hybrid, BIF: Bimolecular Fluorescence Complementation, FRET: Förster resonance energy transfer.

Protein–protein interactions (PPIs) drive all biological systems at the cellular, subcellular and extracellular level, and changes in the specificity and affinity of these interactions may be responsible for cellular malfunctions. Thus, PPIs identification is a key factor for plant biologists to gain knowledge about the relationships among proteins and how they affect biological functions. An important contribution in solving PPIs has been provided by conventional structural techniques, such as X-ray crystallography and NMR, and by yeast two-hybrid (Y2H) assays [173] (Figure 7). In addition to Y2H [174], bimolecular fluorescence complementation (BiFC) and Förster resonance energy transfer (FRET) have been used to establish PPIs in plant proteomes [175]. These approaches provide excellent results but they are limited to small scale experiments. However, FRET-based methods have been also used for high-throughput screening of PPIs using protein microarrays [176].

A more comprehensive identification of PPIs is achieved by combining protein purification strategies with MS-based proteomics [177]. PPIs identified by AP-MS studies are commonly ranked and scored using both computational methods, that predict the most robust and important interactions [178], and quantification approaches, that allow a measure of the importance of these interactions determining the stoichiometry of all of the interactors [179]. Since AP-MS provides also information about indirect protein-protein interactions, these data are especially valuable in combination with results provided by metho ds that show physical PPI, including FRET or cross-linking (XL) methodologies. In particular, advances in cross-linking chemistry and tools for data analysis have promoted cross-linking (XL) in combination with MS as a powerful tool to comprehensively identify PPIs [180]. In addition to the relative simplicity of implementation, the main strengths of XL-MS concern its global character, the capacity to characterize protein complex stoichiometries and topologies. Although XL-MS experiments may be performed near physiological conditions, in vivo studies still suffer some technical difficulties including the limited diversity of cross-linkers, their lower solubility and their cell penetration ability which can be further affected in the case of plants due to their cell wall. Finally, a further general drawback is related to the computational challenges to process XL-MS data; in fact, search engine algorithms have to consider all the possible peptide pair combinations, making the database search engine step time-consuming.

Cross-link enrichment and data analysis have been recently addressed by Zhu et al. to optimize in vivo cross-linking in *Arabidopsis thaliana* [181]. The authors established a MudPIT procedure for the enrichment of cross-linked peptides and developed a bioinformatic tool, called ECL, to an exhaustive cross-linked peptides identification in plant chemical cross-linked peptides. A global investigation of *Arabidopsis thaliana* PPIs was also performed by combining MS with biochemical fractionations e.g., size-exclusion chromatography (SEC), which is suited to separate and identify hundreds of putative complexes, including novel subunits [182]. On the contrary, it seems that to date no studies have adopted perturbation experiments to investigate PPIs in plants. They are based on a variety of assays that analyse the co-behavior of proteins. For instance, thermal proximity coaggregation is based on the hypothesis that interacting proteins co-aggregate upon heat denaturation, leading to similar solubility across different temperatures [183]. This methodology is interesting because it is suitable to study membrane proteins and because it enables the intracellular study of the dynamics of multiple protein complexes simultaneously in intact cell and tissues. However, these methods are labor-intensive and the current inability to distinguish between physical and functional PPIs is a limitation in protein interaction studies. In particular, it is difficult to classify proteins shared by different protein complexes, while complexes that are biochemically similar but functionally distinct are not separated.

As for plant interactome, PPIs are available for *Zea mays*, *Oryza sativa*, *Solanum lycopersicum* and especially for the model plant *Arabidopsis thaliana* (Table 1). Most of these PPIs are computationally predicted and different studies on plant organisms focused on this kind of approach to identify PPIs at the large scale level [36,184,185,186]. Many of the networks computationally predicted have been inferred by transferring the link from orthologs in reference plants. This approach may introduce false positive identification because orthologs are often paralogs, and thus they are functionally divergent. For instance, no more than half of the *Arabidopsis thaliana* coding genes have orthologs in more than 20 of the 27 fully sequenced crop species, suggesting that the associalogs of *Arabidopsis thaliana* network have limited coverage for most crop plants [47]. However, plant gene networks may also benefit from the associalogs (conserved functional linkages transferred from other organisms by orthology) of networks for non-plant species. In fact, many divergent phenotypes between animals and plants have evolutionary conserved gene networks [187]. Thus, given that a limited amount of data derived from plants are available in public databases, the associalogs of non plant gene networks are considered an important resource for crop network inference. On the other hand, the lack on PPIs knowledge about plant organisms is driving many biologists to reconstruct PPI networks experimentally (Table 2). Of note, a number of studies and databases (Table 3) focus on host-pathogen PPI, suggesting that this field of PPIs is of great interest to improve strategies oriented to manage destructive pathogens that cause huge crop losses every year worldwide [35,184,185,188,189,190].

### 3.4. PPI Networks

**Figure 8:**
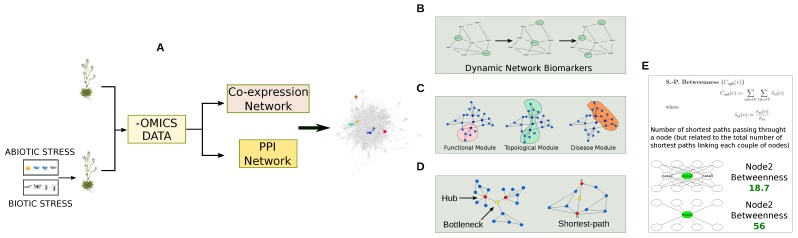
Topological and functional evaluation of co-expression and PPI network models. (**A**) Protein and transcript levels are used to reconstruct co-expression networks or to be combined to PPI networks; (**B**) The reconstructed networks are matched with differentially expressed proteins to characterize dynamical network biomarkers that represent genes/proteins showing time-dependent alterations; (**C**) By using cluster algorithms, topological (nodes highly connected), functional (nodes highly connected and cooperating to carry out a specific function) and disease (nodes highly connected and cooperating in developing a specific disease) modules are identified; (**D**) The network structure is analysed by specific topological parameters (or centralities) [38], including betweenness, to identify hub or bottleneck genes/proteins, while the Shortest Path measure finds all the shortest paths between two nodes; (**E**) Betweenness centrality and simulation of its variation.

Pathways, networks and macromolecular assemblies are commonly represented through PPI networks. They refer to a graph G = (V , E), where a set of nodes V, stands for the proteins, while a set of edges E, stands for their interactions [9], (Figure 7). PPI networks allow for the evaluation of large scale proteomics data taking into consideration functional and physical relationships among proteins. They are usually processed at a functional and topological level. The functional evaluation of PPI or co-expression networks is usually performed by approaches based on the GO term enrichment [155,156]. On the other hand, it is more and more established that network structure is closely related to biological functions. Thus, starting from this point, many studies concerning plants are facing biological questions by investigating network models in terms of topology [44,174,189,191,192,193,194]. As a result, functional, topological and disease modules, as well as hubs, bottleneck and dynamic network biomarkers are new concepts that are impacting the understanding of the processes determining the pathophysiological states of plants (Figure 8).

#### Studies Combining PPIs and Network Topology

To our knowledge, the combination of large scale proteomic data and PPI networks in plant models has been adopted in a very small number of studies, and one of them was conducted by Duan et al. [194]. To investigate the dynamics of protein phosphorylation events due to changes in nutrient conditions, the authors mapped the experimentally identified phosphoproteins on the *Arabidopsis thaliana* PPI network. In addition to identifying the most topologically relevant proteins, dynamic phosphorylation networks were reconstructed and proteins were grouped in “initiation”, “processing” and “effector” layers based on their in- and out-degree level. Following the layering based on network topology, the authors found specific phosphorylation motifs and layer-specific kinases. It suggested that the topology of the inferred networks is highly indicative of an information dissemination architecture in which signals pass through different and well defined layers.

Although PPI networks should be combined with experimentally identified proteins, when protein profiles are unavailable they are often matched with transcript levels. This is the case for Xie et al., who in their study investigated the floral pattern formation in *Arabidopsis thaliana* by using public floral and non-floral gene expression data [192]. Through the combination of the weighted gene co-expression network analysis (WGCNA) and Support Vector Machine (SVM) method, they analyzed gene co-expression network and PPIs. The topological analysis allowed the identification of seven modules and, inside them, the top hub proteins selected by eigengene-based connectivity (kME) and gene significance (GS) parameters. In addition to identifying new genes putatively involved in flower development, the authors elucidate the functions of the floral patterning genes by combining evolutionary information and the *Arabidopsis thaliana* PPI network. In this way, they found that the characterized modules corresponded to the regulation units of flower development.

PPI networks and topology analysis were also used by Zhu et al. to identify novel *Arabidopsis thaliana* genes related to fruit-associated biological processes [44]. The authors applied a shortest-path based method to analyze all shortest paths connecting any two of a set of validated genes. Since the endpoints of these paths were *Arabidopsis thaliana* genes related to fruit biological processes, the authors assumed that genes corresponding to the extracted shortest could also be related to these fruit processes. The strength of this hypothesis was confirmed by the presence of the validated genes in the extracted paths, indicating the effectiveness of the shortest-path based method to select genes involved in the same processes. Of note, the authors took into account the topological structure of the network as a potential factor that could influence their results. In fact, hub proteins can exhibit general associations with many other proteins, so that they are more easily identified by the shortest-path algorithms. As a consequence, since these proteins could have more or less association with the investigated processes, the authors preferred to exclude them from further considerations.

An interesting use of PPI networks in the context of plant biology concerns the understanding of host-pathogen molecular relationships and thus infection processes. This topic was addressed by Bosque et al., who evaluated the effect of the *Potyviridae virus* family on *Arabidopsis thaliana* [189]. PPI networks were reconstructed for several species of potyvirus, and after their topological evaluation (by clustering coefficient, closeness, betweenness and topological coefficient) they were integrated with the complete *Arabidopsis thaliana* PPI network to reconstruct a Plant-Pathogen Interaction Network (PPIN). Starting from each viral protein, and by using a shortest path based method, the authors calculated how many steps were needed to reach each host proteins. In this way, the authors obtained an indication of the anchor points used by the virus to affect the plant PPI network. Interestingly, through the topological analysis it was found that some viral proteins act earlier than others during the infection cycle. In addition, some of them act on host hubs, while others target many proteins affecting the propagation of their effect. Similarly, the investigation of host-pathogen molecular relationships was addressed by Li et al. [191]. Of note, the authors performed an integrative network analysis by combining *Arabidopsis thaliana*.

PPI network, effectors of *Pseudomonas syringae* and *Hyaloperonospora* on *Arabidopsidis thaliana* known targets and gene expression profiles. The topological analysis of the reconstructed networks suggested that the effectors tended to manipulate key network positions with higher betweenness centrality. In addition, effector targets, especially those sharing an individual effector, tended to belong to the same module. The higher betweenness centrality of effector targets partly explained how the local impact due to effectors could have global influences upon the host cellular network. In fact, the authors claimed that effectors employ potent local impact mode to interfere with key positions in the host network and to quickly translocate their effect to all its targets. On the other hand, they found that the plant organizes a defense by sequentially activating genes distal to the effector targets. Of note, pathogen-susceptible mutants tended to have more DEGs surrounding the effector targets compared with resistant mutants, and the distances between the effector targets and DEGs increased over time during infection.

## 4. Discussion and Conclusions

Large scale molecular studies on plant organisms are mainly characterized by genomics and metabolomics, while proteomics is still less widespread and its potential has to be largely explored. This delay is mainly caused by the paucity of proper genomic and protein sequences which are a key factor in performing the MS spectra interpretation by database searching algorithms [213]. In fact, in addition to non-model organism, it is significant that the majority of sequenced crop species are not available in the public databases, thus the number of studies addressing their proteome profiling, including PPIs, are scarce. On the other hand, studies reporting proteome profiles counting thousands of proteins are often limited in their functional evaluation due also to the lack of proper annotations, including gene ontology terms. For these reasons, data-derived systems biology approaches to investigate plant organisms are dominated by strategies that evaluate transcriptomic data in the form of co-expression networks. These studies have in common the topological evaluation of the reconstructed network in driving the identification of new candidate genes. It is interesting that to improve this procedure of selection some authors combined molecular profiles and population-based data provided by GWAS and QTL studies [19,51,52]. This strategy was also recently adopted to identify functional associations between genes and metabolism in *Arabidopsis thaliana* [15,16,17], while no studies, to our knowledge, have so far included high-throughput proteomic data. However, the integration of complementary approaches and data is mandatory to improve the quality of plant research, and despite all the limitations related to plant organisms, the results reported in the proteomic studies mentioned here are promising and they are attracting the interest of a growing number of plant biologists. In fact, different examples demonstrated the power of high-throughput proteomic technologies to address plant biology questions by characterizing thousands of proteins per sample and multiple biomarkers [23,25,26,27,28,29,30].

Although the large amount of data produced by proteomic experiments well blend with data-derived systems biology approaches, greater efforts must be made regarding the characterization of the intra- and extra-cellular PPIs. While a very low number of studies combined large scale proteomic data and PPI networks to investigate the molecular mechanisms underlying plant phenotypes, PPIs are gaining great interest in investigating plant-pathogen relationships. Every year pathogenic attacks cause billions of dollars’ worth of damage to crops and livestock, thus to reconstruct the PPI network between host and pathogen may represent a plus in elucidating the molecular basis of the pathogenesis and to improve defense strategies [13]. In this scenario, the above-mentioned studies highlighted the great effectiveness of the network topological analysis in identifying the anchor points that pathogens use to affect the plant network, as well as elucidating the mechanisms of plant response [189,191]. In addition to hub analysis, these studies have further emphasized the potential of other topological measures, such as the shortest path, to find a correlation between topology and biological functions. Of note, similar concepts were successfully proposed by Barabasi et al. to predict the therapeutic and side effects of several drugs, the similarity of action between different drugs and the possibility to repropose the use of existing drugs [11].

Globally, the results discussed in this review confirm the informative power that lurks inside the network structure, both PPIs and co-expression. Thus, we expect to see in the next few years an exponential increase of studies combining the application of high-throughput proteomic technologies and systems biology approaches to investigate plant organisms. However, in particular for proteomic studies, a gap must be filled. The discovery and quantification power of shotgun and targeted proteomic approaches will have to be combined to analyze in depth the proteome of plant organs, cell types and subcellular compartments. At the same time, it will be crucial to increase experimental studies to characterize PPIs and network models highly complete, reliable and specific. In fact, although great efforts have been made to computationally predict PPIs, these approaches unfortunately are associated with the risk of a high rate of false positive identification. These goals surely represent the major challenges to be faced in the near future by plant biologists and their achievement cannot be separated from their synergistic cooperation with other biologists, physicists, mathematicians and bioinformatics.

## Figures and Tables

**Table 1:** Databases collecting predicted and experimentally validated PPIs in plant organisms. E: Experimentally validated, P: Computationally predicted.

Database	db Link	Proteins	PPIs	Organism
MIND	http://biodb.lumc.edu/mind/index.php	-	12,102 (E)	*A. thaliana*
PAIR	http://www.cls.zju.edu.cn/pair/home.pair	-	329,044 (P) and 6257 (E)	*A. thaliana*
AI1	http://signal.salk.edu/interactome/AI1.html	2774	6205 (P)	*A. thaliana*
SUBA	http://suba.live/	-	19,933 (P)	*A. thaliana*
AraPPINet	http://netbio.sjtu.edu.cn/arappinet/	12,574	316,747 (P)	*A. thaliana*
AtPID	http://www.megabionet.org/ atpid/webfile/index.php	5562	28,062 (P)	*A. thaliana*
AtPIN	http://bioinfo.esalq.usp.br/atpin	-	96,000 (E/P)	*A. thaliana*
PRIN	http://bis.zju.edu.cn/prin/	5049	76,585 (P)	*O. sativa*
DIPOS	http://comp-sysbio.org/dipos/	27,746	14,614,067 (P)	*O. sativa*
RicePPINet	http://netbio.sjtu.edu.cn/riceppinet/	16,895	708,819 (P)	*O. sativa*
PPIM	http://comp-sysbio.org/ppim/	14,000	2,762,560 (P)	*Z. mays*
PiZeaM		6004	49,026 (P)	*Z. mays*
PTIR	http://bdg.hfut.edu.cn/ptir/	10,626	357,946 (P)	*S. lycopersicum*
MauPIR	http://14.139.59.222:8080/MauPIR/	1812	6804 (P)	*Macrotyloma uniflorum*
PlaNet	http://bml.sbi.kmutt.ac.th/ppi/index.php	7209	90,173 (P)	*Manihot esculenta*
*Citrus sinensis* Annotation project	http://citrus.hzau.edu.cn/orange/index.php	8195	124,491 (P)	Fruit crop
FPPI	http://comp-sysbio.org/fppi/python/Default_PredictedPPIs.html	7406	223,166 (P)	*Gibberella zeae*

**Table 2:** Studies focused on PPIs detection, both experimentally validated (Exp. val.) computationally predicted (Comp. pred.), in plant organisms.

Organisms	Proteins	PPIs	Topic	Exp. Val.	Comp. Pred.	Ref.
*A. thaliana*	25,123	-	Fruit development	Yes	-	[185]
*A. thaliana*	5598	13,328	Immune response	Yes	-	[195]
*A. thaliana*	3056	18,233	Immune response	Yes	-	[196]
*A. thaliana*	2355	25,172	Network topology	Yes	-	[197]
*A. thaliana*	393	857	Cell cycle	Yes	-	[198]
*A. thaliana*	13,136	42,131	Network analysis/Methods	-	Yes	[199]
*A. thaliana*	13,347	45,058	Cell-wall synthesis	-	Yes	[200]
*A. thaliana*/*Pseudomonas syringae*	-	14,043 (*A. thaliana*)	Host-pathogen interaction	-	Yes	[35]
		1337 (*Pseudomonas syringae*)				
*A. thaliana*/*Ralstonia solanacearum*	1442 (*A. thaliana*)	3074	Host-pathogen interaction	-	Yes	[201]
	119 (*Ralstonia solanacearum*)					
*A. thaliana*/*Potyvirus*	5127 (*A. thaliana*)	12,624	Host-pathogen interaction	-	-	[189]
	11 (*Potyvirus*)					
*O. sativa*	16,895	708,819	Network inference/Methods	-	Yes	[186]
*O. sativa*	5049	76,585	Network inference/Methods	-	Yes	[202]
*O. sativa* subsp. indica	454	4114	Abiotic stress response	-	Yes	[203]
*O. sativa*/*Ustilaginoidea virens*	3305	20,217	Host-pathogen interaction	-	Yes	[204]
*O. sativa*/*Rhizoctonia solani*	1773	6705	Host-pathogen interaction	Yes	-	[184]
*Macrotyloma uniflorum*	1812	6804	Abiotic stress response	-	Yes	[205]
*Z. mays*	14,000	2,762,560	Network inference/Methods	-	Yes	[206]
*Citrus sinensis*	8195	124,491	Network inference/Methods	-	Yes	[207]
*Gleditsia sinensis*	1897	7078	Network inference/Abiotic stress response	Yes	-	[208]
*Tetraselmis subcordiformis*	938	12,887	Network inference/Metabolism	Yes	-	[209]
*Manihot esculenta*	7209	90,173	Network inference/Methods	-	Yes	[210]
*Pinus taeda*/*Sirex noctilio*	528	4363	Host-pathogen interaction	Yes	-	[211]
apple, maize, pear, rice, strawberry and tomato/*Penicillium expansum*	9911	439,904	Host-pathogen interaction	-	Yes	[212]

**Table 3:** Databases collecting host-pathogen PPIs, including plants.

Database	db Link	Proteins	PPIs	Organisms
PPIRA	http://protein.cau.edu.cn/ppira/	-	3074	*R. solanacearum* and *A. thaliana*
PPIN-1	http://signal.salk.edu/interactome/PPIN1.html	926	3148	*Pseudomonas syringae* and *A. thaliana*
PathoPlant	http://www.pathoplant.de/	-	350	Plants and plant-pathogen
PHI-base	http://www.phi-base.org/	-	8046	Multispecies host-pathogen
HPIDB	http://hpidb.igbb.msstate.edu/	-	62,653	Multispecies host-pathogen
VirHostnet	http://virhostnet.prabi.fr/	-	28,000	Multispecies host-virus
VirusMentha	http://virusmentha.uniroma2.it/	4426	9876	Multispecies host-virus
EBI Intact	https://www.ebi.ac.uk/intact/	105,180	805,177	Multispecies host-pathogen

## Supplementary Materials

The following are available online at http://www.mdpi.com/2227-7382/6/2/27/s1, Table S1: Representative list of bioinformatic tools to assist researchers in -omic data processing, from profiling to networking.

## Abbreviations

The following abbreviations are used in this manuscript:

2DE	2 Dimensional Gel Electrophoresis
2D-DIGE	2 Dimensional Fluorescence Difference Gel Electrophoresis
AP-MS	Affinity Purification Mass Spectrometry
AQUA	Absolute Quantification
BiFC	Bimolecular Fluorescence Complementation
CAM	Crassulacean Acid Metabolism
CE	Capillary Electrophoresis
DDA	Data Dependent Acquisition
DEGs	Differentially Expressed Genes
DEPs	Differentially Expressed Proteins
DIA	Data Independent Acquisition
emPAI	Exponentially Modified Protein Abundance Index
FlexiQuant	Full-Length Expressed Stable Isotope-labeled Proteins for Quantification
FT-MS	Fourier Transform-Mass Spectrometry
ELISA	Enzyme-Linked Immunosorbent Assay
FRET	Förster Resonance Energy Transfer
GC	Gas Chromatography
GO	Gene Ontology
GRN	Gene Regulatory Network
GS	Gene Significance
GWAS	Genome Wide Association Study
HILIC	Hydrophilic Interaction Liquid Chromatography
HMDB	Human Metabolome Database
HPLC	High Performance Liquid Chromatography
ICAT	Isotope-Coded Affinity Tag
ICPL	Isotope-Coded Protein Label
kME	Eigengene-Based Connectivity
LC	Liquid Chromatography
MeRy-B	Metabolomic Repository Bordeaux
miRNA	microRNA
MRM	Multiple-Reaction Monitoring
mRNA	messenger RNA
MS	Mass Spectrometry
MS/MS	tandem mass spectra
MudPIT	Multidimensional Protein Identification Technology
MW	Molecular Weight
NGS	Next Generation Sequencing
NMR	Nuclear Magnetic Resonance
NSAF	Normalized spectral abundance factor
ODEs	Ordinary Differential Equations
PC	Pearson’s Correlation
PCS	Peptide-Concatenated Standards
pI	Isoelectric point
PMN	Plant Metabolic Network Database
PPI	Protein-Protein Interaction
PPIN	Plant-Pathogen Interaction Network
PRM	Parallel Reaction Monitoring
PTMs	Post Translational Modifications
PrEST	Protein Epitope Signature Tag
QconCAT	Quantification Concatemer
QTL	Quantitative Trait Locus
RNA-Seq	RNA Sequencing
siRNA	small interference RNA
SpC	Spectral Count
SEC	Size-Exclusion Chromatography
SNP	Single Nucleotide Polymorphism
SRM	Selected Reaction Monitoring
SVM	Support Vector Machine
SWATH	Sequential Windowed Acquisition of All Theoretical Fragment Ion Mass Spectra
SWIM	SWItchMiner
TAP-MS	Tandem Affinity Purification coupled with Mass Spectrometry
TMT	Tandem Mass Tags
WGCNA	Weighted Gene Co-expression Network Analysis
XICs	Extracted Ion Chromatogram
XL	Cross Linking
Y2H	Yeast Two Hybrid
